# The distribution and mutagenesis of short coding INDELs from 1,128 whole exomes

**DOI:** 10.1186/s12864-015-1333-7

**Published:** 2015-02-28

**Authors:** Danny Challis, Lilian Antunes, Erik Garrison, Eric Banks, Uday S Evani, Donna Muzny, Ryan Poplin, Richard A Gibbs, Gabor Marth, Fuli Yu

**Affiliations:** Human Genome Sequencing Center, Baylor College of Medicine, Houston, TX 77030 USA; Department of Molecular and Human Genetics, Baylor College of Medicine, Houston, TX 77030 USA; Present address: Monsanto Company, Ankeny, IA 50021 USA; Present address: Washington University School of Medicine, Saint Louis, MO 63110 USA; Department of Biology, Boston College, Wellcome Trust Sanger Institute, Chestnut Hill, MA 02467 USA; Program in Medical and Population Genetics, Broad Institute of Harvard and MIT, Cambridge, MA 02142 USA; Present address: New York Genome Center, New York, NY 10013 USA; Present address: Department of Human Genetics and Utah Center for Genetic Discovery, University of Utah School of Medicine, Salt Lake City, UT 84112 USA; Institute of Neurology, Tianjin Medical University General Hospital, Tianjin, 300052 China

**Keywords:** INDEL, 1000 Genomes Project, Distribution, Mutagenesis

## Abstract

**Background:**

Identifying insertion/deletion polymorphisms (INDELs) with high confidence has been intrinsically challenging in short-read sequencing data. Here we report our approach for improving INDEL calling accuracy by using a machine learning algorithm to combine call sets generated with three independent methods, and by leveraging the strengths of each individual pipeline. Utilizing this approach, we generated a consensus exome INDEL call set from a large dataset generated by the 1000 Genomes Project (1000G), maximizing both the sensitivity and the specificity of the calls.

**Results:**

This consensus exome INDEL call set features 7,210 INDELs, from 1,128 individuals across 13 populations included in the 1000 Genomes Phase 1 dataset, with a false discovery rate (FDR) of about 7.0%.

**Conclusions:**

In our study we further characterize the patterns and distributions of these exonic INDELs with respect to density, allele length, and site frequency spectrum, as well as the potential mutagenic mechanisms of coding INDELs in humans.

**Electronic supplementary material:**

The online version of this article (doi:10.1186/s12864-015-1333-7) contains supplementary material, which is available to authorized users.

## Background

The difficulties in identifying true INDELs in coding exomes are threefold: (1) next-generation sequencing (NGS) methods are prone to produce INDEL artifacts [1], (2) mapping can be problematic [2,3], and (3) low INDEL density in the coding regions of the genome results in a very low signal to noise ratio (that is, the ratio between the number of true INDEL loci versus the number of sequencing and alignment artifacts is low) [4,5]. Many tools and analysis pipeline have been developed for the purpose of identifying indels in NGS data [6,7]. However, previous studies have demonstrated that the different exome variant calling pipelines produce indel call sets with very low concordance (less than 30%) [8].

The 1000G aims to provide a comprehensive resource of human genetic variation throughout the world by sequencing the whole genomes (with low coverage) and exomes (with high coverage) of 2,500 individuals from diverse global populations [4,5]. In Phase 1 of the project, exome sequencing data of 1,128 individuals was generated and released. In our study we generated a consensus call of the exome INDELs from these 1,128 individuals and characterized the INDELs in terms of density, allele length and frequency, selection patterns, and mechanisms of mutagenesis.

## Results

### Generating a high quality consensus call set from three different call sets

Mapping and INDEL calling in the 1,128 exomes from the 1000G [5] were initially performed using multiple pipelines (i.e. Atlas2 [9,10], FreeBayes [11] and GATK-UnifiedGenotyper [2]) by three different groups (see Methods, Additional file 1: Figure S1). The different call sets were merged into a union call set with 14,611 candidate INDEL loci (Additional file 1: Figure S2). Next, through our consensus procedure using a random forest model (Methods), we identified 7,210 short (<100 base pairs) coding INDELs (Table 1). This is considered as our consensus call set and based upon for further analysis in this study. The random forest model evaluated the strength of evidence for each individual INDEL allele based on a series of parameters, e.g. the number of supporting call sets, read-based quality metrics, and the normalized average of the INDEL qualities reported by the different variant callers (see Methods, Additional file 1: Table S1). Most of these parameters have been reported by the calling pipelines directly in their variant call outputs (in variant call format or VCF files), making it unnecessary to re-process the original BAMs when making the consensus call set, rendering the consensus generation procedure very efficient. After the consensus call set i.e. the sites of INDEL variants were generated, each sample was re-genotyped at each variant site (Additional file 1: Figure S1), by going back to the primary sequence alignment (or BAM) file.

**Table 1:** **Summary statistics of the consensus INDEL call set from the exomes of the 1000 Genomes Project**

	**Continental group**				**Total merged**
	**Africa**	**Americas**	**Asia**	**Europe**	
# Samples	250	199	293	386	1128
# INDEL loci	2689	2057	2334	2555	7210
% Novel (not in low coverage)	78.0	71.5	84.7	81.6	90.03
% Rare (AAF < 1%)	68.1	71.3	78.3	79.3	96.7
# INDELs per exome	173	146	133	142	147
% frameshift INDELs per exome	44.1	40.3	40.1	41.9	41.7

**Figure 1:**
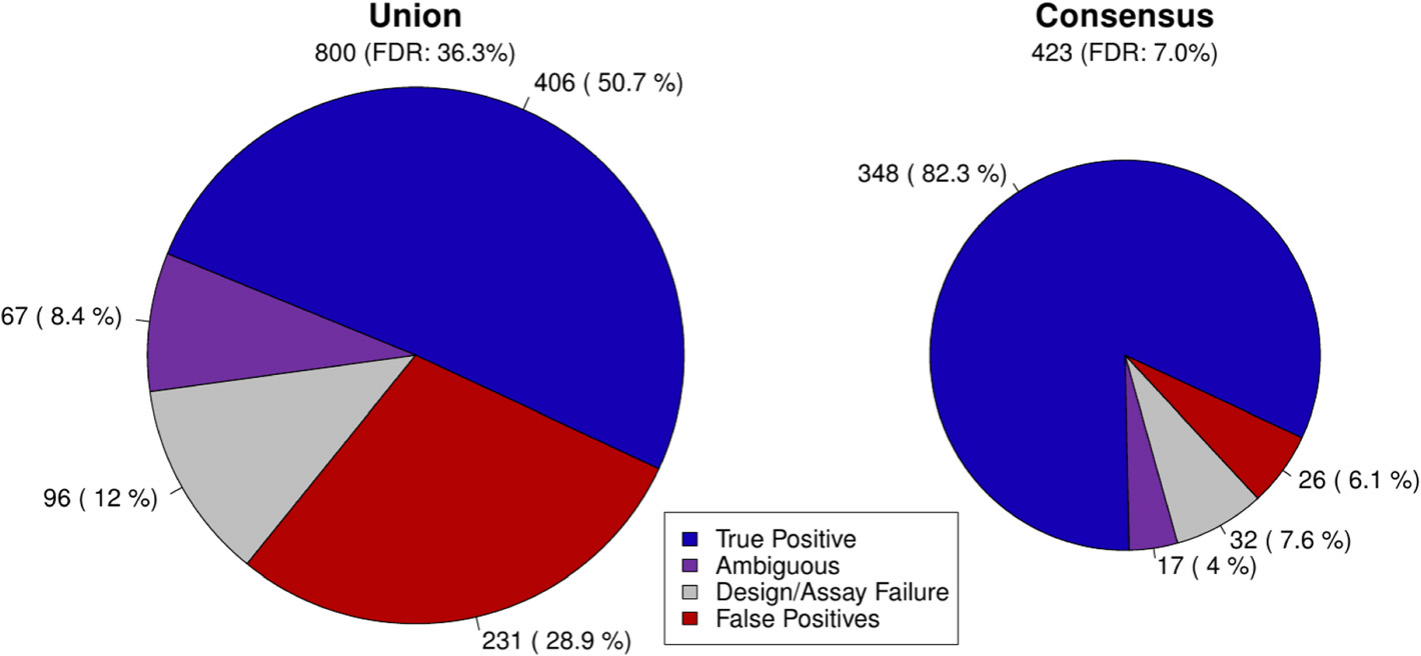
**Validation results at population level for both the initial union call set and the consensus call set.** The population validation experiment involved validating 800 randomly selected INDEL sites in up to 5 individuals. Among the validated loci, 423 loci were in our consensus call set. The consensus process lowers the estimated FDR by 29.3%.

To evaluate the quality of the consensus call set versus the initial union set, 800 INDELs were randomly selected from the union call set (by allele frequency) and submitted for PCR Roche 454 validation (see Methods). On the basis of the validation results, we estimated an FDR of 7.0% and 36% for the consensus call set and the union call set respectively (Figure 1). However, the consensus call set lost 14% of the true INDELs in the union set. On a single-sample level validation of two individuals, we estimated FDRs of 9.2% and 10.8% (Additional file 1: Figure S3), respectively.

A second validation was also performed confirming the exact loci and alleles of the consensus set INDELs using the Illumina HiSeq and MiSeq platforms at high depth coverage on two individuals. This validation estimated an FDR of 7.5% and 4.4% and confirmed over 99% of the true positives from the Roche 454 validation experiment (see Methods).

We also compared the consensus call set to the 1000G low-coverage whole-genome Phase 1 dataset (i.e. low coverage whole genome data in the same samples where we examined deep coverage exome sequences), and found that 90.03% of the INDELs we found were novel (Table 1). This can be attributed to the fact that coding exome INDELs are predominately rare, with 96.7% having an alternative allele frequency (AAF) less than 1%, and therefore are inaccessible to low coverage sequencing [4,12]. Our exome-based consensus INDEL call set had an average of 1.5 times more deletions than insertions per- individual exome. In contrast, the deletion to insertion ratio in the 1000G low-coverage dataset was 1.7 [13]; the similar ratios indicate that the surplus of deletions is not due to DNA capture issues in the exome data, but more likely due to the difficulty of mapping short reads containing insertions.

### Negative Darwinian selection against coding exome INDELs

We also observed that selection pressure against coding INDELs is notably higher than SNPs, resulting in reduced INDEL density. We calculated the average variant density per individual (Table 2, see Methods), and found an exome INDEL density of 5.52 INDELs per mega-base (Mb), which is 118 times lower than the exome SNP density, and 24 times lower than the whole genome INDEL density calculated from the 1000G Phase 1 data [5].

**Table 2:** **The variant density per individual is compared for exome INDELs, exome SNPs and whole genome INDELs from the 1000 Genomes Project**

	**Average density per individual (INDELs/Mb)**	**Genomic region**
Exome INDELs	5.5	1000G exome
Exome SNPs	649.4	1000G exome
Non-coding INDELs	134.7	1000G whole genome (without exome region)
HuRef exome INDELs	4.1	1000G exome
YanHuang exome INDELs*	2.6	1000G exome

For exome INDELs the consensus set is also compared to the HuRef and YanHuang call sets. Variant density was calculated by dividing the average variants per individual by the size of the analyzed exome target region or non-coding genome as indicated. *The YanHuang exome only includes INDELs of length 3 bp or less.

**Figure 2:**
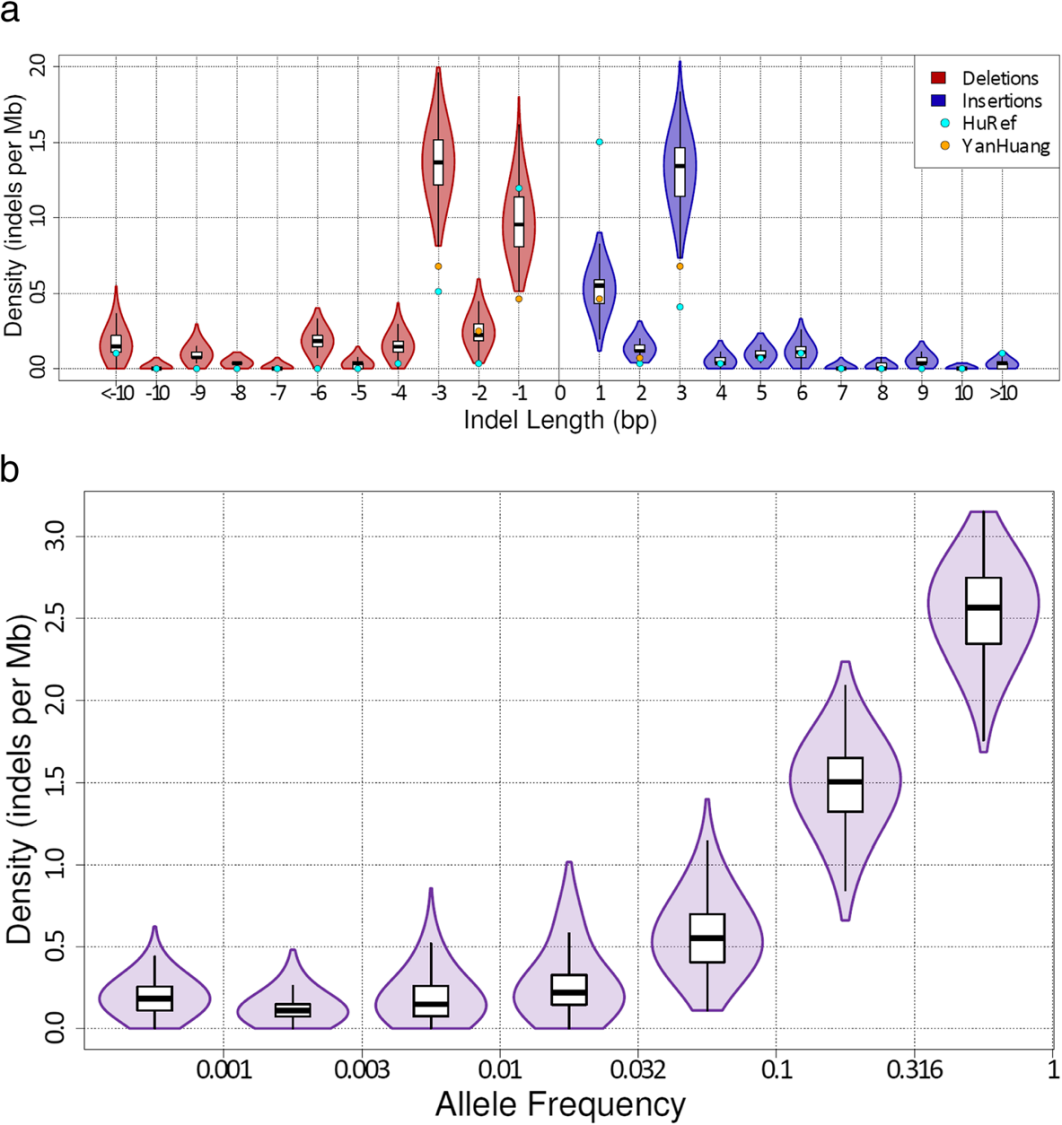
**Exome INDEL call set analysis per individual. a**. To characterize the exome INDEL length distribution in individuals we calculated the density of exome INDELs of different lengths in each individual. Shorter INDELs are generally common with a strong bias towards INDEL lengths that are a multiple of 3 (inframe). The INDEL density for the HuRef and YanHuang exome are also shown for reference. Deletions are shown with negative length. **b**. To characterize the allele frequency distribution in individuals we calculated the exome INDEL density in each individual across 7 logarithmic allele frequency bins. The results confirm that most of the exome INDELs in an individual are common (>10%), but most individuals harbor a small number of extremely rare exome INDELs (i.e. singletons).

Frameshift INDELs (INDEL length is not multiple of three bases) are under especially strong scrutiny as they generally result in a nonsense mutation and changes in amino acid sequences. In order to further characterize this effect we evaluated the distribution of INDEL lengths (Figure 2a). In our call set we discovered INDELs ranging in size from −84 to 12 base pairs (deletion lengths are represented as negative numbers). The vast majority (95%) of the INDELs are short (<10 base-pairs), and the distribution is strongly enriched for in-frame INDELs (i.e. where INDEL length is a multiple of three bases) (39%). Short INDELs were the most frequent with an average of 1.50 1 bp-INDELs per Mb of sequence, and an average of 0.19 large (>10 bp) INDELs per Mb (Figure 2a). At the individual exome level, selection against frameshift INDELs is even more pronounced, with an average of 2.31 frameshift INDELs per Mb, and an average of 3.21 inframe INDELs per Mb. In fact, larger in-frame INDELs are observed more frequently than shorter frameshift INDELs. These findings are directly related to the power law of INDEL mutagenesis and clearly demonstrate selection against frameshift INDELs.

We calculated the INDEL density of each individual across different allele frequencies to characterize the allele frequency spectrum in individuals (Figure 2b). The results confirmed that protein coding INDELs are indeed predominantly common on the individual level, with an individual harboring an average of 4.03 INDELs per Mb with an allele frequency of 10% or greater, 0.86 INDELs per Mb with an allele frequency between 1% and 10%, and 0.50 INDELs per Mb with an allele frequency less than 1%. When comparing the population level distribution of allele frequency on exome INDELs, low-coverage whole genome SNPs, and INDELs and exome SNPs (Additional file 1: Figure S4), we found that the exome INDELs and frameshift INDELs in particular are rarer than SNPs or non-coding INDELs, with a significantly lower minor allele frequency (MAF) (p < 2.2e-16, Wilcoxon Rank Sum test)

### Polymerase slippage is the predominant mechanism of exome INDEL mutagenesis

**Figure 3:**
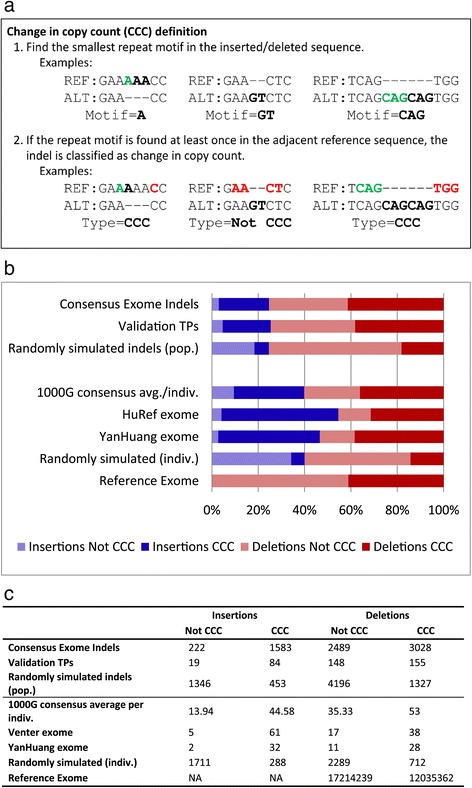
**CCC analysis. a**. All INDELs in the analysis we classified as either change in copy count (CCC) or not change in copy count (NCCC) as defined in the figure. **b**. The CCC vs NCCC percent distribution of insertions (blue) and deletions (red) was calculated at both the population level (row 1) and the individuals level (row 4). The distributions were also compared against validation results, random simulations (see Methods), HuRef, YanHuang, and the human reference genome (deletions only). **c**. The counts of CCC vs NCCC distribution in insertions and deletions.

One of the well-known mechanisms of INDEL mutations is the occurrence of polymerase slippage during replication which generally results in the expansion or contraction of tandem repeat regions [14]. In this analyses, INDELs related to the mechanism of polymerase slippage are classified as change in copy count (CCC) INDELs, where the smallest repeat motif of the INDEL represents either an expansion or a contraction of the adjacent reference base or bases (Figure 3a). To estimate what proportion of the INDELs were likely formed through polymerase slippage events, we classified each of the INDELs as either CCC or not-CCC (NCCC) (Figure 3b).

We discovered that the majority of INDELs (63.0%) in our consensus call set are CCC and therefore likely caused by polymerase slippage. The FDR is expected to be higher in lower-complexity regions, but the low overall FDR (estimated at 7%) means this is true even if all the FPs are CCC. When we further classified the INDELs into insertion and deletion, we found that the proportions of CCC insertions and deletions were 21.6% to 41.4% (significantly higher P < 0.0001) respectively. And the fractions of NCCC insertion to deletion in the consensus dataset were 3.0% to 34.0%, respectively. This represents a seven-fold enrichment of CCC insertion events than NCCC insertion events. While insertions are known to be more difficult to map and identify in short-read sequencing data, the NCCC insertions are less repetitive and should be easier to discover than CCC insertions. These findings show that polymerase slippage or similar repeat-based events are the mechanism for nearly all insertions.

The prevalence of CCC INDELs, and CCC insertions in particular, has been observed in a previous study which analyzed CCC INDELs at the whole genome level [13], but in order to further establish that they are not due to technical or analytical artifacts, we performed this same analysis on the INDELs from our population validation experiment, the HuRef [15] and YanHuang [16] exomes, and on randomly generated INDEL call sets based on the consensus set’s length distribution (see Methods). The validation experiment, the HuRef and Yan Huang analyses all show the same lack of NCCC insertions, indicating that the effect is not due to technical or analytical artifacts. The randomly simulated data sets serve as controls to show what is expected to occur by chance for an INDEL call set with the given length distribution and exome target region.

Even the small percentage of NCCC insertions (3.0%) may be explained by complex events with underlying CCC disrupted by SNPs. If complex events were the majority of NCCC insertions, we would expect them to be rarer in population and more prevalent at the individual level. The time for complex events to occur and disrupt the repeat pattern is more recent. We calculated the average number of CCC and NCCC INDELs per individual in the call set and confirmed that the average percent of CCC insertions is indeed higher (23.8%) than the NCCC insertion ratio (11.5%).

If indeed the NCCC insertions were derived from CCC mutagenesis initially, we would also expect NCCC insertions to have characteristics (such as frameshift rate) similar to those characteristics of the CCC INDELs. To test this we calculated the average frameshift rate for CCC and NCCC insertions and deletions. For the insertions we confirmed that the NCCC frameshift rate is in fact very similar (within 2%) to CCC insertions (Additional file 1: Table S2), consistent with the hypothesis that most of the NCCC insertions were originally CCC insertions. Deletions on the other hand had an elevated frameshift rate in NCCC deletions (12.3% higher than CCC). The higher frameshift rate suggests that some mechanism other than polymerase slippage is more likely involved in generating frameshift NCCC deletions.

## Discussion

In this study we have identified 7,210 coding INDELs in a diverse global population of 1,128 individuals. By leveraging the high depth of coverage afforded by exome capture technology we are able to identify more rare variants than is possible through low coverage sequence. Using multiple independent INDEL calling pipelines with machine learning based consensus model algorithm to produce a consensus call set we were able to maximize the consensus call set’s sensitivity and specificity. The validation of 800 INDELs across the population and by individual level demonstrated that the consensus call set is of high quality with an FDR of approximately 7%.

As expected, the consensus call set shows that coding exome INDELs are under strong selection compared to SNPs and non-coding INDELs. This is evident in the overall density where exome INDELs have a significantly lower density than the other variant types. It is also apparent in the allele frequency distributions, with exome INDELs having significantly lower MAFs than the other variant types. The selection is also apparent at the individual level as there are very few rare (and thus more likely damaging) INDELs in each individual. Frameshift INDELs are clearly shown to be under even greater selection pressure as their density is significantly lower than in-frame INDELs and the length distribution violates the power law expected for INDEL mutagenesis.

## Conclusions

Previous studies have shown that INDELs more commonly occur in repeat regions or as CCC INDELs in the human genome, possibly by polymerase slippage mechanism [13]. While long repeat regions are rare in the exome, our findings confirm that there is significant enrichment of CCC INDELs, indicating that polymerase slippage is a primary driver of INDEL mutagenesis in the human exome. Furthermore, our data provides evidence that nearly all exome insertions are the result of polymerase slippage events, including most of the NCCC insertions.

One limitation of this study is that the findings are drawn from an INDEL calling pipeline which was not designed specifically for detection of CCC or repeat region INDELs. Future analysis using variant calling pipelines more specialized for these repeat-based INDELs [17,18] will likely reveal additional insights on their mutagenesis and other characteristics.

## Methods

### INDEL calling

Sequencing data was obtained from the 1000 Genomes Phase 1 exome project [12]. Additional post-processing mapping and INDEL calling was performed independently at the Baylor College of Medicine (BCM), Boston College (BC) and the Broad Institute (BI).

### BCM INDEL calling pipeline

The HGSC-BCM INDEL calling pipeline was run on all 1,128 samples in the 1000G Phase 1 Exome project using the officially released BAM files (MOSAIK [19] mappings for Illumina, BFAST [20] mapping for SOLiD). The SOLiD BAM files were locally realigned using the GATK local realignment utility [2]. The Illumina BAM files have already undergone local realignment as part of the 1000G production pipeline. INDEL calls were made on each individual using the Atlas2 variant analysis suite [9,10]. The resulting variant call format (VCF) files were then merged into a population VCF where non-variant depth and genotypes were filled in.

### BC INDEL calling pipeline

The BC INDEL calling pipeline was run on all 1,128 samples in the 1000G Phase 1 Exome project using the officially released BAM files. In order to ensure consistent representation of INDELs, both SOLiD and Illumina BAM files were realigned at runtime using the left alignment utility in the FreeBayes variant caller package (bamleftalign), and were split in cases where mismatches could be resolved by a larger insertion or deletion using gap-opening realignment (ogap) [11]. INDEL, SNP, and complex variant calls were generated simultaneously using the FreeBayes variant detector. Subsequently, loci containing INDELs were extracted using utilities in the vcflib VCF manipulation suite.

### BI INDEL calling pipeline

The BI INDEL calling pipeline was run on only the 822 Illumina samples in 1000G Phase 1 Exome project using the officially released FASTQ files mapped using the BWA aligner [21]. BAM files were locally-realigned and the base-qualities recalibrated using GATK. INDEL calls were then made using the GATK Unified Genotyper [2].

### Union call Set

Each of these call sets was merged into a union set after complex events were split into simple INDEL events and left aligning the INDELs. This merging, like all other analyses in this study, was limited to autosomal chromosomes. The union set was regenotyped using the Atlas2 genotyping method and the BAM files used in the HGSC-BCM INDEL calling pipeline. Rather than the official 1000G BAM files BC used an alternative mapping method. For INDELs unique to the alternative mappings, the genotypes reported in the original call set were used. This union was annotated to indicate which centers called each INDEL, what INDEL quality scores were reported, and what other metrics we considered likely to be useful for generating a high quality consensus.

### Random forest model

The Random Forest model was developed by calculating and collecting data for 31 covariates from the union set at loci in samples that overlapped with calls made using the Complete Genomics (CGI) analysis pipeline. For the purpose of training the model, INDELs from the union set that were confirmed by the CGI data were considered true positives and those not confirmed were considered false positives. The Affymetrix Axiom exome genotyping array [22] was also tested as a standard, but did not perform as well (data not shown).

From this training set a variety of machine-learning methods were evaluated based on sensitivity and precision (Additional file 1: Figure S6). The random forest methods were found to be most effective. The random forest method was selected as the final method because of its nearly equivalent performance and much faster runtime. Covariates that were determined to be too biased, redundant, or ineffective were removed from the random forest model. The final consensus model includes the 6 covariates described in Additional file 1: Table S1.

### Consensus call Set

Using the random forest model, we generated a probability of being a true INDEL for each INDEL allele. INDEL alleles with a probability less than 0.4 were not included in the final consensus set. To make the indel genotypes consistent, the final consensus set was regenotyped, and genotype likelihoods were calculated using a modified version of the latest Atlas-INDEL2 genotyper. This changed the genotype of individuals and in some cases the genotype was determined to be homozygous reference. After regenotyping, INDELs which were no longer called in any individuals were removed from the call set.

### Variant density, allele length and frequency

The average variant density and percent heterozygous were calculated for the consensus exome INDEL set and compared against the exome SNPs and non-coding INDELs of the 1000G phase 1 integrated call set. These values were calculated for each individual in the call sets, and then averaged. The variant density was calculated by dividing the number of called variants by the number of base-pairs in the analysis. For the exome SNPs and INDELs this region was the autosomal region of the consensus exome capture target used in phase 1 of 1000G [ftp://ftp.1000genomes.ebi.ac.uk/vol1/ftp/phase1/analysis_results/supporting/exome_pull_down/20110225.called_exome_targets.consensus.bed]. For the exome INDELs, which did not undergo imputation analysis, we further limited this region to bases with a read depth of at least one (region was calculated independently for each individual). For the he HuRef and YanHuang exomes the same 1000G exome target region was used. For non-coding INDELs the size of the accessible genome as reported in the 1000 Genomes Phase 1 publication [5] was used excluding the consensus exome capture target. This same analysis was repeated for each INDEL length category (Figure 2a) and for seven alternative allele frequency bins (Figure 2b). Within these same regions the percent heterozygous was calculated by dividing the number of heterozygous variants by the total number of called variants in each individual, and then taking the average.

The minor allele frequency (MAF) was calculated for the consensus exome INDEL call set and compared against the MAFs of the official 1000G phase 1 whole genome SNP and INDEL call sets (Additional file 1: Figure S4). We also filtered the whole-genome SNP call set to the 1000G phase 1 consensus exome capture target region to include the exome SNPs in the comparison. In addition, we split the consensus exome INDEL call set into frameshift and inframe call sets and calculated the MAF distribution for each of these. In comparing all these MAF distributions we used the Wilcoxon Rank Sum test as a test of significant difference.

### In silico validation

*In silico* validation of the 1000G phase 1 exome INDEL consensus and the union sets was performed by comparison to INDELs obtained from 1000 Genomes phase 1 low coverage [ftp://ftp.1000genomes.ebi.ac.uk/vol1/ftp/phase1/analysis_results/integrated_call_sets/] and whole exome INDEL genotypes from Affymetrix Axiom Genotyping Solution [ftp://ftp.1000genomes.ebi.ac.uk/vol1/ftp/phase1/analysis_results/supporting/axiom_genotypes].

The analysis was performed on a single individual level by comparing each overlapping individual separately between the call sets. All INDELs were left aligned and filtered to restrict the comparison to protein coding regions as defined in the 1000G phase 1 exome project [ftp://ftp.trace.ncbi.nih.gov/1000genomes/ftp/phase1/analysis_results/supporting/exome_pull_down/20110225.called_exome_targets.consensus.bed]. INDELs were compared on the basis of the genomic position. For every overlapping individual, confirmation and rediscovery rate were determined and the average individual confirmation and rediscovery rate across all overlapping individuals was calculated. Confirmation rate is defined as total number of INDELs in consensus or union set matching an INDEL in the validation set. Rediscovery rate is the number of INDELs called in the validation set matching an INDEL in the consensus or union set.

### Experimental validation

Experimental validation was performed using the HGSC-BCM PCR-Roche 454 INDEL validation pipeline. The validation included both a population level experiment and an individual level experiment. For the population validation experiment, 800 INDEL sites were randomly selected from the union set using GATK to preserve the allele frequency distribution. For each site up to five individuals that have variant were randomly selected for validation (fewer if less than 5 were variants). The individual validation experiment was performed on two samples: NA19238 and NA10851. We validated all INDEL sites that were designated as variant in these two samples in the union set.

Once the validation site and samples were selected, primers were designed using the Primer3 based HGSC-BCM Primer Pipeline. After PCR amplification and normalization the DNA was sequenced on the Roche 454 sequencing platform. After the sequenced reads were mapped to the human reference genome (Build 37) with BLAT [23], the mapped reads were aligned to the amplicon sequence using CrossMatch [24]. INDELs identified in the aligned reads were considered matching if they were within 30 bp of the original INDEL (5 bp for 1 bp INDELs) and of the exact same INDEL length. In order to be considered confirmed, the variant read ratio (number of reads with the INDEL divided by the total read depth) had to be at least 20% (40% for 1 bp INDELs) and the average base quality had to be at least 10 (20 for 1 bp INDELs). INDELs with a variant read ratio less than 3% (5% for 1 bp INDELs) are considered false positives. INDELs with variant read ratio between these values are considered ambiguous. If the site failed in primary design, or PCR, or if there were fewer than 20 reads covering the site, the validation was considered a failure, and no conclusions were drawn.

A follow up validation was also performed confirming the exact loci and alleles of the consensus set indels in two individuals. This validation was performed using both the HiSeq and MiSeq Illumina sequencers at the Broad Institute. Analysis-ready BAM files were generated for both MiSeq and HiSeq with the best practices data processing pipeline (BWA alignment, Picard’s Mark Duplicates, GATK’s Base Quality Score Recalibration, and GATK’s Indel Realignment).

The consensus set indel calls were passed as input alleles to GATK’s Unified Genotyper in “Genotype Given Alleles” mode. In that mode the genotyper does not do discovery but rather attempts to genotype the provided alleles against the information in the BAM file. The alleles are genotyped exactly as is (in the position with the exact base composition). All discrepant (the 2 techs disagreed) or monomorphic (the 2 techs agreed against the consensus calls) sites were manually confirmed by visual inspection in Integrative Genomic Viewer (IGV) [25].

### Validation analysis

Once the validation results were received, the FDR was estimated as follows:

$$ FDR=\frac{TP}{TP+FP} $$

The validated results were then subset to only include INDEL sites included in the consensus set, and this subset was used to estimate the consensus set FDR (Figure 1). Additionally, the consensus set’s relative sensitivity (*S*_*consensus*_) or rediscovery rate was calculated by dividing the number of consensus TPs by the number of union TPs.

$$ {S}_{consensus}= relative\_ sensitivity=\frac{T{P}_{consensus}}{T{P}_{union}} $$

For the individual validation we also estimated the actual number of INDELs detectable in the data. This was done by summing the number of INDELs confirmed in the union set, the number of consensus ambiguous/failed sites times one minus the consensus FDR, and the number of consensus ambiguous/failed sites times one minus the consensus rediscovery rate.

$$ detectable\_ indels=T{P}_{union}+\left(AMB{G}_{conensus}+FAI{L}_{consensus}\right)*\left(1-{S}_{consensus}\right) $$

### Ethical considerations

As this study analyzes only publicly available data, there are no additional ethical concerns to be considered beyond those addressed in the publications connected to the original data release [4,5,22].

## Availability of supporting data

Consensus call set available for download at:

ftp://ftp.1000genomes.ebi.ac.uk/vol1/ftp/technical/working/20121024_phase1_exome_indel_regenotyped/ALL.phase1_exome_indel_consensus_regenotyped_withAA.20110521.indels.exome.genotypes.vcf.gz

## Additional file

Additional file 1: **Supplementary Material.**

## Abbreviations

INDEL: Insertions and deletions
1000G: the 1000 Genomes Project
FDR: False discovery rate
NGS: Next generation sequencing
BAM: Binary version of a SAM (sequence alignment/map) format file
MAF: Minor allele frequency
AAF: Alternative allele frequency
Mb: Megabases
CCC: Change in copy count indels (see Figure 3a)
NCCC: Not change in copy count INDELs
TP: True positive
FP: False positive
