# Supplementary material for: The distribution and mutagenesis of short coding INDELs from 1,128 whole exomes
Source: BMC Genomics. 2015 Feb 28;16(1):143. doi: 10.1186/s12864-015-1333-7 (PMC4352271; doi:10.1186/s12864-015-1333-7)
Supplement: Supplementary file 1 — Supplementary Material. [file 12864_2015_1333_MOESM1_ESM.docx]

# Supplementary Material

**In Silico Validation**

To understand the quality of the consensus indel calls, we compared it with other data sets generated using orthogonal technologies (Supplementary Table 3, see Methods), including the 1000 Genomes phase 1 low-coverage indel call set (prior to final filtering, 1039 samples) [5] and the Affymetrix Axiom Genotyping Solution (903 samples) [22]. On average 64.2% are confirmed by 1000G Phase 1 low-coverage data and 59.3% are confirmed by the Affymetrix Axiom chip. The comparison also showed that the consensus call set rediscovered 57.4% of the 1000G low-coverage calls and 19.1% of the Affymetrix Axiom chip calls. The very low rediscovery rate for the Axiom chip suggests that the consensus set has low sensitivity. However, the union set also has a very low rediscovery rate of 28.2%, indicating that the Axiom chip results contain many false positives which are lowering this rate.

**Validation details**

The first validation experiment involved randomly selecting 800 indel sites from the union in a manner that maintained the allele frequency distribution (i.e. population indel validation). The second experiment included all indels from the union called in two individuals (i.e. individual indel validation): NA19238, which is from the Yoruba in Ibadan, Nigeria (YRI) population and was sequenced on the Illumina platform, and NA10851, which is from the Utah residents with Northern and Western European ancestry (CEU) population and was sequenced on the SOLiD platform. We selected these two samples attempting to understand the dependencies of indel ratio with respect to the ethnicity and technologies.

The population validation study results illustrate that the consensus method was able to drastically reduce the false discovery rate (FDR) from 63.1% to 7.1%, while still retaining 84.2% of the true indels (Figure 1). Evaluating the random forest model with the validation data confirms that the selected probability cutoff effectively maximizes the consensus call set’s sensitivity and specificity (Supplementary Figure 5). 20% of the validated sites returned an ambiguous result or failed to produce a result in the validation experiment. These sites were further analyzed by comparison with indel calls on overlapping samples made using the Affymetrics Axiom chip and the 1000G low-coverage data (see Supplementary Table 4). In addition, the consensus set indel sites for NA10851 and NA19238 were resequenced using the Illumina HiSeq and MiSeq platforms at high depth coverage (see Methods, Supplementary Table 5). This data confirmed 92.5% and 95.6% of the indels called in NA10851 and NA19238 respectively, with 80% and 90% of the sites with ambiguous or failed validation results being confirmed as indel polymorphisms – considered true positives.

From the “individual validation” experiment we discovered that NA19238 has at least 189 short coding indels and NA10851 has at least 137 (Supplementary Figure 3). Based on the number of ambiguous/failed indels in the consensus set and compensating for limited sensitivity (see Methods), we estimate that NA19238 actually has about 254 short coding indels detectable on this platform and NA10851 actually has about 181. The individual validation study also demonstrates the effectiveness of the consensus method at removing false positives, reducing the FDR from 24.7% to 9.2% in NA19238 and from 63.1% to 10.8% in NA10851.

1bp indels are a particularly difficult indel class to deal with because they are the indel size that is most common in the genome and are also by far the most common false indel caused by sequencing or alignment errors, meaning that often either sensitivity or precision must be sacrificed due to the low signal to noise ratio. Our analysis discovered an average of 40 (27%) 1bp indels per sample (Figure 2a). Besides the 1bp indels, we compared a large range of indel length distributions for the consensus set and found the consensus indel sets show clear peaks at non-frameshift causing indel lengths.

The validation results for the consensus call set were also analyzed by allele frequency. It was found that singleton and rare indels had the lowest false discovery rate (FDR), while low-frequency and common indels had the highest (Supplementary Table S6). It should be noted that the Illumina HiSeq/MiSeq validation indicates that the PCR-Roche 454 validation overestimates FDR. Table S5 shows that in two individuals 77% and 85% of the indels which were classified as false indels in the 454 validation were actually confirmed in the HiSeq/MiSeq validation.

The higher error rate in common indels likely reflects both the error mode of the read mapping and the limitations of the variant calling methods. Incorrect mapping which produces false indels is likely to occur in multiple individuals since they often share a common haplotype in the region and are being mapped to the same reference genome by the same method. The result is a common indel with relatively high allele frequency. Rare false indels are generally the results of sequencing read errors which the variant calling methods are good at identifying.

# Supplementary Figures

## Figure S1 – Pipeline Summary


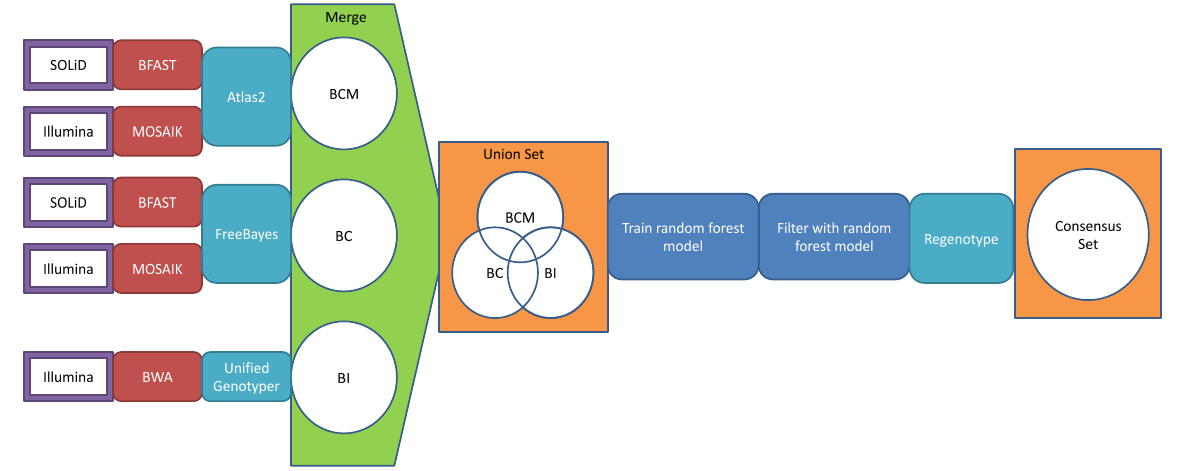


## Figure S2 – Indel Union set

## Figure S3 – Validation Results

## Figure S4 – Allele Frequency


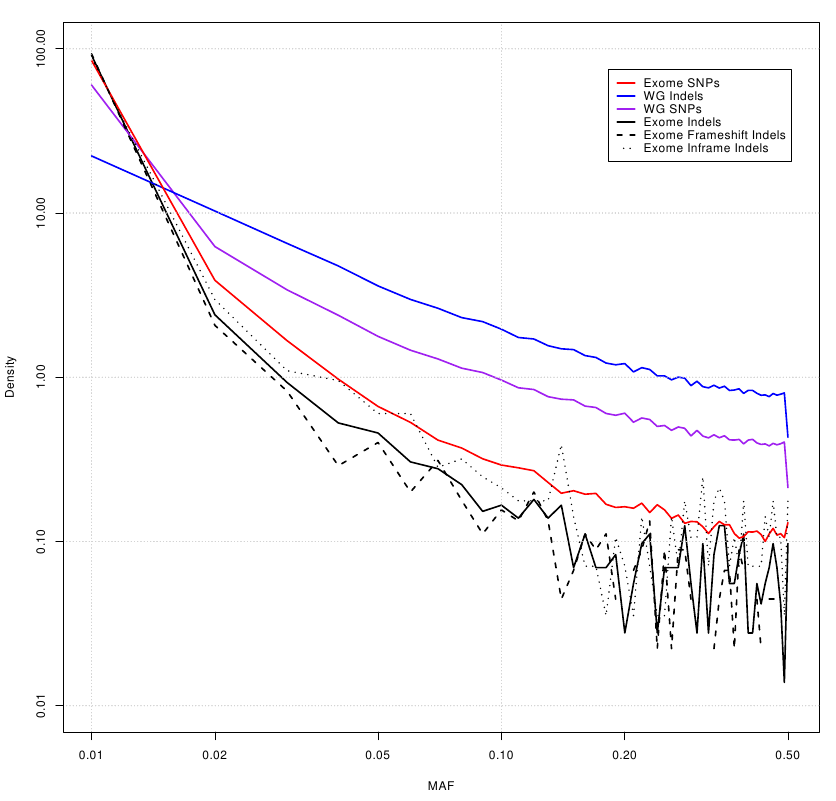


## Figure S5 – Consensus model evaluation

## Figure S6 – Machine learning method comparisons


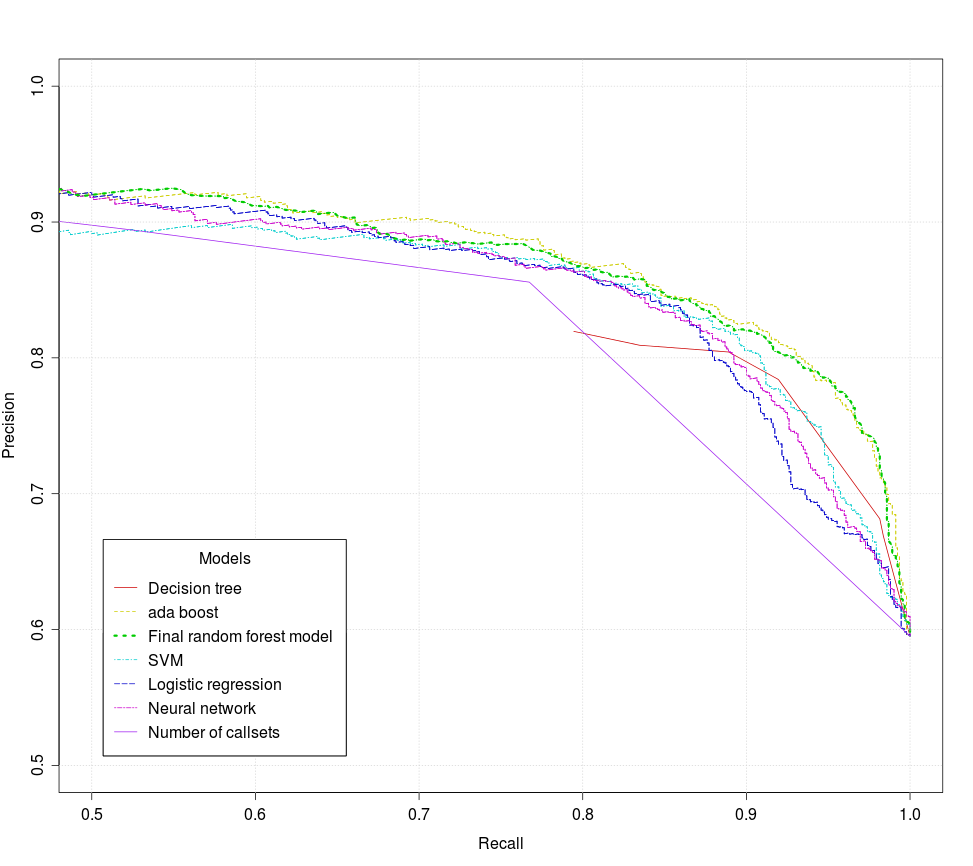


# Supplementary Tables

## Table S1: Consensus Model Covariates

| **Covariate** | **Description** |
| --- | --- |
| Supporting call sets ratio | # of sets indel called in/# possible (based on sequencing platform) |
| Average normalized variant square | Variant reads2 / total reads: averaged across all variant samples |
| Samples per allele | # of samples with at least one variant read / Allele count |
| Normalized average indel quality | Average of the normalized indel quality score reported in each call set |
| Single strand ratio | Ratio of variant samples with variant reads all in one strand direction |
| Average variation rate | Average number of other variants on variant reads / read length: averaged across all variant samples |

## Table S2 – Average frameshift rate of insertions and deletions in CCC and NCCC indels

|  | Insertions  (% frameshift per individual) | Deletions  (% frameshift per individual) |
| --- | --- | --- |
| All exome indels | 35.47 | 40.06 |
| CCC exome indels | 35.10 | 41.94 |
| NCCC exome indels | 36.72 | 54.22 |

## Table S3: In Silico Validation results

| **Other Call Sets** | **Overlapping Samples** |  | **Confirmation Rate** | | **Rediscovery Rate** | |
| --- | --- | --- | --- | --- | --- | --- |
|  |  |  | **Illumina** | **SOLiD** | **Illumina** | **SOLiD** |
| **Lowcov** | **1039** | **Consensus** | 64.22 | 61.18 | 57.44 | 59.76 |
|  |  | **Union** | 27.21 | 19.68 | 64.98 | 64.49 |
| **Affy** | **903** | **Consensus** | 59.33 | 60.25 | 19.10 | 21.65 |
|  |  | **Union** | 32.55 | 25.48 | 28.16 | 30.72 |

## Table S4. % overlap for AMBG and FAIL validation results

|  |  | **True Positives** | **False Positives** | **AMBG** | **Consensus AMBG** | **Uncalled AMBG** | **FAIL** | **Consensus FAIL** | **Uncalled FAIL** |
| --- | --- | --- | --- | --- | --- | --- | --- | --- | --- |
|  | Low-Pass | 57.66 | 2.14 | 10.20 | 60.00 | 4.55 | 20.54 | 51.43 | 6.49 |
|  | Affy | 61.31 | 6.84 | 32.65 | 60.00 | 29.55 | 19.64 | 42.86 | 9.09 |
|  | Low-Pass | NAN | NAN | NAN | NAN | NAN | NAN | NAN | NAN |
|  | Affy | 53.97 | 1.61 | 29.73 | 60.00 | 25.00 | 23.90 | 50.00 | 16.26 |

## Table S5. % of PCR-Roche 454 validation results confirmed by Illumina HiSeq/MiSeq

|  | **True Positives** | **False Positives** | **AMBG** | **FAIL** | **Total** |
| --- | --- | --- | --- | --- | --- |
| NA10851 | 99.07 | 84.62 | 80.00 | 80.00 | 92.55 |
| NA19238 | 99.22 | 76.92 | 100.00 | 88.89 | 95.60 |

Table S6. PCR-Roch 454 validation results by allele frequency

|  | **Total** | **Confirmed** | **Non confirmed** | **ambiguous** | **assay fail** | **Design fail** | **FDR** |
| --- | --- | --- | --- | --- | --- | --- | --- |
| Total | 423 | 348 | 26 | 17 | 3 | 29 | 0.0695 |
| singletons | 252 | 219 | 6 | 11 | 1 | 15 | 0.0267 |
| Rare <1% | 130 | 101 | 14 | 6 | 2 | 7 | 0.1217 |
| Low Freq 1-5% | 21 | 12 | 2 | 0 | 0 | 7 | 0.1429 |
| Common >5% | 20 | 16 | 4 | 0 | 0 | 0 | 0.2000 |
